# Cost and cost-effectiveness of health behavior change interventions implemented with self-help groups in Bihar, India

**DOI:** 10.1371/journal.pone.0213723

**Published:** 2019-03-28

**Authors:** S. Chandrashekar, S. Saha, B. Varghese, L. Mohan, G. Shetty, A. Porwal, A. Hazra, S. Mondal, R. Das

**Affiliations:** 1 Karnataka Health Promotion Trust, Bangalore, India; 2 Public Health Foundation of India, New Delhi, India; 3 Indian Institute of Public Health, Gandhinagar, India; 4 Population Council, New Delhi, India; 5 Project Concern International, New Delhi, India; Gettysburg College, UNITED STATES

## Abstract

**Introduction:**

Health interventions implemented with self-help groups (SHGs) enhance the relevance and acceptability of the health services. The Parivartan program was implemented in eight districts of Bihar with women’s self-help groups to increase adoption of maternal and newborn health behaviors through layering health behavior change communication. This study estimates the cost and cost-effectiveness of a health behavior change program with SHGs in Bihar.

**Methods:**

Cost analysis was conducted from a provider’s perspective. All costs have been presented in US dollars for the purpose of international comparisons and converted to constant values. The effectiveness estimate was based on the reported changes in select newborn care practices. A decision model approach was used to estimate the potential number of neonatal deaths averted based on adoption of key newborn care practices. Using India’s life expectancy of 65 years, cost per life year saved was calculated. A one-way sensitivity analysis was conducted using the upper and lower estimates for various variables in the model, and functionality of SHGs.

**Results:**

The cost of forming an SHG group was US$254 and that of reaching a woman within the group was US$19. The unit cost for delivering health interventions through the Parivartan program was US$148 per group and US$11 per woman reached. During an 18 months period, Parivartan program reached around 17,120 SHGs and an estimated 20,544 pregnant women resulting in an estimated prevention of 23 neonatal deaths at a cost of US$3,825 per life year saved.

**Conclusion:**

SHGs can be an effective platform to increase uptake of women’s health interventions and follow-up care, and also to broaden their utility beyond microfinance, particularly when they operate at a larger scale.

## Introduction

Self-help groups (SHGs) are informal groups of 10–20 individuals, mostly women and usually from the same village, formed with the objective of promoting collective savings. In recent years, there have been experiments and other efforts to engage SHGs to increase access to health services and to build bridges between the formal health systems and the communities to which they belong. It is argued that health interventions implemented through SHGs can enhance the uptake of health services.

Programs that promote women’s access to credit and livelihood have modest, but positive gains for women and households [1]. Women’s increased access to resources have positive associations with improved health such as immunization rates, reduce child mortality, and result in smaller family size and greater well-being of families [2, 3].

A meta-analysis of seven trials conducted in low resource settings found the introduction of community-based interventions towards improving maternal and newborn care through engagement of women’s groups was associated with the following:

- a 37% reduction in maternal mortality (odds ratio: 0·63, 95% CI: 0·32–0·94),
- a 23% reduction in neonatal mortality (odds ratio: 0·77, 95% CI :0·65–0·90),
- a 9% non-significant reduction in stillbirths (odds ratio: 0·91, 95% CI :0·79–1·03) [4].

In Maharashtra *Mahila Aarogya Samiti*, or women’s health groups, were established to address the problems of large population size, limited healthcare resources, and healthcare accessibility. Early evidence from this innovation suggests that this initiative has generated increased awareness about the importance of antenatal care, immunization, and institutional deliveries and enhanced the *Janani Suraksha Yojana* (JSY, a conditional cash transfer program) uptake among community women [5]. In Kerala SHG participation was found to help protect poor women against exclusion from health care and could possibly aid in promoting their mental health [6]. Another study analyzed national-level data from the third District-Level Household Survey from 601 districts in India to assess the influence of the presence of SHGs in a village on maternal health service uptake. The study showed respondents from villages with an SHG were:

- 19% more likely (OR: 1.19, CI: 1.13–1.24) to have delivered in an institution,
- 8% more likely (OR: 1.08, CI: 1.05–1.14) to feed colostrum to newborns,
- 19% more likely (OR: 1.19, CI: 1.11–1.27) to utilize family planning products and services [7].

Studies on the costs of formation and promotion of SHGs are limited. Harper (2002) provided some of the early estimates of the costs of SHG promotion [8]. That study reported that the costs of developing an SHG from start to bank linkage were found to range between US$21 and US$246, and noted that cost depends on existing levels of cohesion within the community. Tankha (2002) computed estimates of the costs of promotion of SHGs of 10 leading NGOs and projects [9]. The estimates varied from US$69 to US$385. UNDP–South Asia Poverty Alleviation Project estimated the cost of promoting an SHG to be US$231; World Bank estimated this figure to be US$308 in Velugu project of Andhra Pradesh [10,11].

The Parivartan program included mobilizing women into self-help groups and adding on health behavior change communication focused on reproductive, maternal, and newborn health, which we call layering. The main aim of this study is to assess the cost effectiveness in terms of cost per neonatal death averted and life year saved resulting from phase 1 of the program.

### Study settings

Parivartan program was rolled out in 55 blocks in eight districts of Bihar and reached 275,000 women of reproductive age through 17,120 SHGs. The program aimed at increasing adoption of key family health and sanitation behaviors through participatory learnings in group settings. The key behaviors being promoted pertain to antenatal care, birth preparedness, postpartum and newborn care, exclusive breastfeeding, complementary feeding, immunization, family planning, and sanitation. In addition to promoting these healthy behaviors, Parivartan aimed to improve access to health care services by facilitating linkages with front-line health care workers. The program in Bihar was designed to establish proof of concept, and was limited to working with a defined number of groups, and reaching women in those groups.

## Methods

### Cost estimation

A cost analysis exercise was undertaken from a provider’s perspective using an ingredients cost approach. The incremental cost of health layering was estimated based on actual expenditure data. To understand the context of delivering intervention activities, data were collected from 2013 to March 2016. Source of information and data included financial records from project implementing partners, project proposals, special studies and narrative annual reports. Field-based sources included interviews and observation of program staff at different levels during field visits, telephone calls, self-administered questionnaires and e-mail exchanges. Retrospectively, staff in the project, such as project managers, regional staff, block-level staff, SHG members, and community resource persons, were asked to estimate the amount of time spent on various activities of the intervention through time sheets.

Capital costs were estimated by including all equipment, furniture, vehicles and start-up costs (until program was rolled out, such as recruitment, office set-up, induction training) used in the program. Each capital item had a fixed useful life, the cost was annualized with a 3% discount. Fixed costs were included, such as cost of development of guidelines for implementation and their adherence, training costs for both staff and facilitators of women’s self-help groups, communication material cost, supplies for mothers and counseling costs.

Costs related to project evaluation and research were excluded from the analysis. Recurrent costs, such as personnel, travel, operating costs, training and indirect management costs, and fixed costs are summed up to give the total cost.

Costs were collected retrospectively and considered both the financial costs and the economic costs. Financial cost represents the actual expenditure on goods and services purchased. Economic cost includes the estimated value of goods and services for which there was no financial transaction. Additional items include donated goods and services such as volunteer labor. This measures the opportunity cost. All costs have been presented in US dollars for the purpose of international comparisons and converted to constant values using prevailing inflation and exchange rates.

Activity-based cost was assessed using time sheets. The budget and expenditure statements were reviewed in order to obtain the costs related to specific activities. Group formation costs included are the following:

- staff costs based on time-sheet data including paid volunteers,
- training costs including development and printing of modules,
- meeting costs,
- costs of nurturing activities, establishing linkages with advocacy and enabling environment activities.

The costs of setting up and maintaining SHGs were calculated where new groups were formed. In blocks where health layering was added to existing groups, incremental costs were calculated from a provider’s perspective. The incremental costs include total costs minus group formation costs and inputs such as module roll-out, training and support activities to enable access to health care are included.

Health layering costs included are staff costs allocated based on time-sheet data including paid volunteers (*sahelis*) in Bihar. In addition, training costs including modules cost, IEC materials, meeting costs, were added to fixed costs of capital items and general expenses over 3 years. Thus, the total costs were calculated for specific activities like capacity building, health layering activities, group formation and nurturing, and knowledge management.

Directly allocable recurrent costs (specific training costs, group formation costs, development and roll-out of specific modules, and personnel responsible for an identified activity) were allocated. Capital costs were allocated equally to all activities. Total costs of the intervention and average costs per process output were calculated using the costs and program output data.

### Effect estimate

The effectiveness estimate was based on the reported changes in select newborn care indicators that were obtained from a quasi-experimental evaluation survey conducted by the Population Council in the Parivartan blocks comparing women members in SHGs with and without the health layering intervention providing coverage information on the above mentioned health indicators. Surveys were conducted at two periods: baseline survey during 2013 and end line survey during 2016. The evaluation surveys were designed to measure changes in key newborn health practices. Neonatal mortality was not measured directly.

The study protocol was reviewed and approved by the Population Council Institutional Review Board. The survey collected coded information on identifiers such as district code, block code, village code, and SHG code, and therefore the data did not include any names, phone numbers, addresses etc. through which respondent’s identity could be revealed. No photographs of the respondents were taken. Informed consent was taken from each respondent before participating in the survey. The research investigator read the consent prepared in the local language, Hindi, explaining the purpose of the study, risks and benefits, confidentiality, voluntariness and rights, compensation, use of study findings and ethical approval of the study. After answering any questions that a respondent might have and confirming her willingness to participate in the survey, the investigator requested a signature on the consent form. If a participant was willing to participate but either could not write or did not want to sign, it is noted as ‘oral consent given’ in the consent form. The present study is a subset of a larger evaluation, and the consent form included permission to share findings through publication in research journals. A copy of the consent form was given to each respondent who agreed to participate in the survey.

Two key systematic reviews of community-level newborn interventions across the continuum of care identified critical indicators that have an impact on neonatal mortality [12,13]. Both studies identified skilled birth attendant, prevention and management of hypothermia (delayed bathing and skin-to-skin care), and initiation of breastfeeding within an hour of delivery as important newborn practices that have an impact on neonatal mortality. From the list of indicators available from the survey, we chose evidence-based practices that have been reported to have a direct link to neonatal mortality. A composite index was then generated: This is based on the fact that, although individual practices have independent effects, the composite index would have much enhanced effect due to the dynamics of interaction as some practices can enhance and increase the adoption of related practices.

**Composite Index.** Delayed bathing of the newborn for 24 hours OR skin-to-skin care AND breastfed within 1 hour of birth AND institutional delivery AND 3 or more ANC visits OR visited by Frontline worker (FLW) within 2 days of delivery OR clean cord care [obtained from mothers with children 0–5 months.

**Fig 1:**
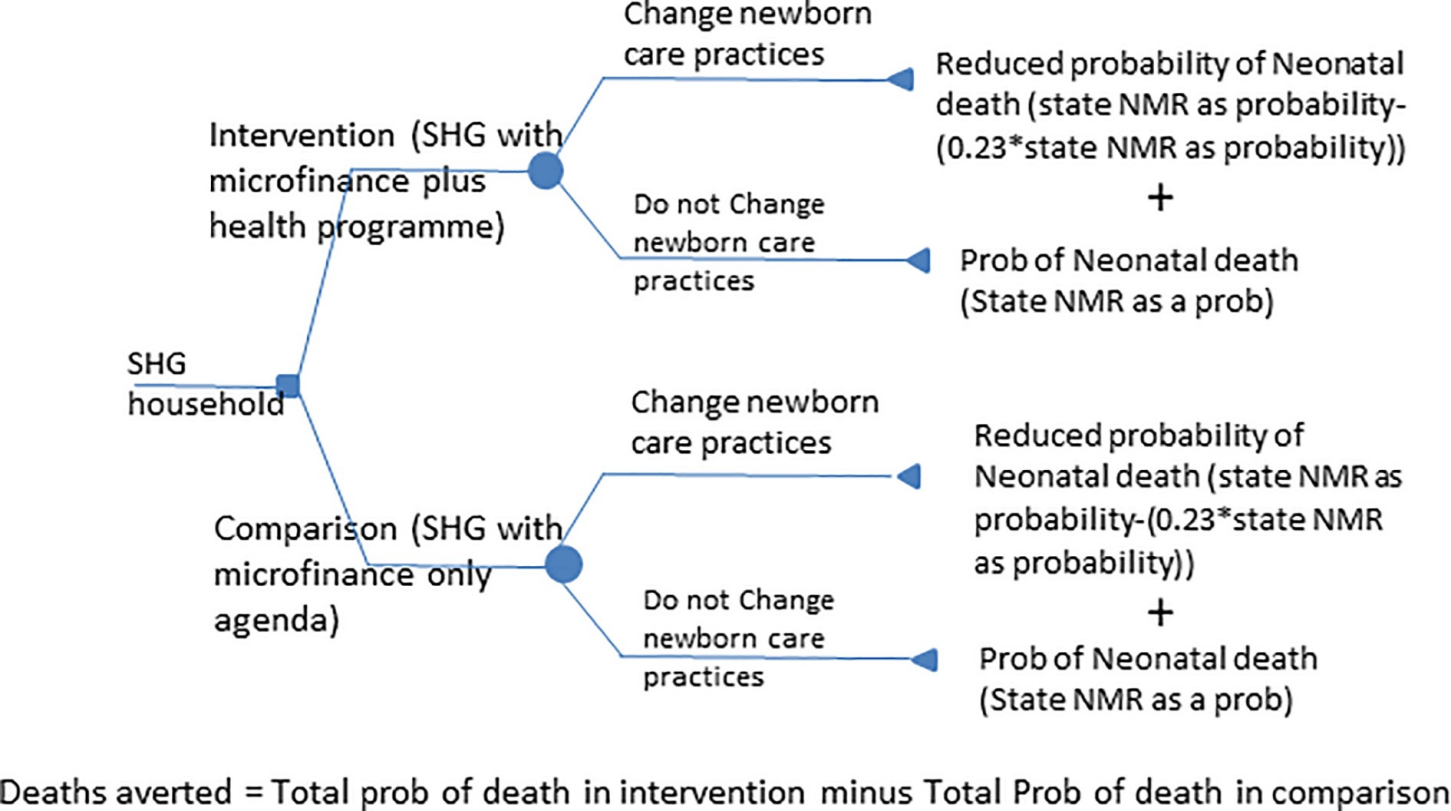
Decision model approach to estimate potential neonatal deaths averted.

We then used a decision model approach (Fig 1) to estimate the potential number of neonatal deaths averted based on adoption of these key practices. Decision modeling is a widely used technique in economic evaluation of programs [14–16]. It is based on the premise of estimating the probability of events such as death or changes in health occurring as a result of choices among possible pathways. Prost *et al*. (2013) in their review of the effectiveness of various community-based interventions among recently delivered women who participated in various group activities [4] provided a pooled estimate of 23% reduction neonatal mortality rate (NMR) from 7 different randomized controlled trials. This pooled estimate for reduction in neonatal mortality rate (23% (95% CI: 10% to 35%)) is used in the decision model to estimate the change in composite index of practices that is associated with the intervention. Table 1 lists the variables that were used to compute the program effect.

**Table 1:** List of variables used in the decision model (for base case).

Variables	Parivartan program	Source
Neonatal mortality (per 1000 live births)	32	Annual Health Surveys 2012–2013 [17]
Reduction in neonatal deaths	23%	Prost *et al*. (2013) [4]
Changes in behavior—composite index	Baseline Endline	Population Council’s Survey [18]
Comparison	19% 36%	
Intervention	17% 50%	

As baseline and end line estimates of maternal and newborn practices were available for intervention and comparison groups, we used a difference-in-differences (difference of two differences) method to estimate the proportion who change newborn care practices.

The women who reported this combination of practices in intervention groups (due to health intervention and exposure to routine health care services) and in comparison groups (due to exposure to routine health care services) were assigned reduced probability of neonatal mortality (23% reduction of neonatal mortality rate (NMR)). Those who did not report this combination of practices were assigned the state-level average NMR (32 per 1000 for Bihar). Thus, the reported differences in maternal and newborn care practices among comparison and intervention groups were the basis for the reported differences in probability of neonatal death among the intervention and comparison groups (Fig 1). Admittedly, this method underestimates the true benefits for mother as potential morbidity reduction is perhaps not fully captured.

The number of neonatal deaths in a group (intervention or comparison) was estimated by the following formula:

(NMR as probability x proportion who do not change newborn care practices) + (reduced probability of neonatal death * proportion who change newborn care practices) multiplied by number of pregnant women in a group.

The estimated number of pregnant women per SHG as determined from field-level estimate was 1.2 [18]. This number was used to assess pregnant women per SHG. Deaths averted due to the intervention were estimated by taking the difference in number of deaths between intervention and comparison groups. Cost-effectiveness ratios were estimated by dividing the incremental cost of the intervention with the number of deaths averted to estimate the cost per neonatal death averted. Although our overall cost estimates included costs of the health layering component, for this analysis we used a conservative estimate of costs. The total cost excluding the costs of group formation has been treated as incremental costs for the health layering intervention.

To estimate the number of life years saved by averting the neonatal deaths, a life expectancy at birth of 65 years multiplied by the number of deaths averted was used. Cost per life year saved was calculated by dividing incremental cost by life years saved. A one-way sensitivity analysis was conducted using the upper and lower estimates for various variables in the model and functionality of SHGs. Functionality of the groups was based on weekly meetings, book of records and weekly saving as revealed from the cross-sectional survey. The baseline and end line surveys indicated that 90% of SHGs were functional.

## Findings

### Cost of program

The average number of members per SHG was obtained from the program data. The cost of forming an SHG group in Bihar was US$254 and that of reaching a woman within the group was US$19 (Table 2). The cost of group formation lies in the middle of the cost range assessed in India [9]. The unit cost for delivering the health layering through the Parivartan model in Bihar was US$148 per group and US$11 per woman reached.

**Table 2:** Cost of parivartan program.

Intervention	Unit cost per SHG (in US$)	Total no. of women reached	Unit cost per woman reached (in US$)
Total Parivartan program cost (n = 17,120 SHGs)	684	338,419	35
Group formation cost in Parivartan program	254	224,287	19
Health layering cost in Parivartan program	148	224,287	11

Note: In addition to group formation and health layering, other cost item included capacity building cost. This is not shown in the table.

### Cost-effectiveness analysis

The Parivartan program for health behavior change communication with SHGs reached around 17,120 SHGs (considering 90% groups were functional) and an estimated 20,544 pregnant women resulting in an estimated prevention of 23 neonatal deaths. This translates to a cost of US$3,825 per life year saved under the base case assumptions (Table 3). Sensitivity analyses showed a range of US$3,221 to US$11,731 per life year saved either with increased coverage of practices or reduction in the cost of the program, or decrease in number of functional SHGs, or higher reduction in neonatal mortality (Table 4).

**Table 3:** Cost-effectiveness analysis.

Variables	Parivartan program
Number of SHGs reached	17,120
Number of pregnant women	20,544
Neonatal deaths averted	23
Cost per neonatal death averted	US$248,650
Cost per life year saved	US$3,825

**Table 4:** Sensitivity analysis for cost per life year saved.

Variables	Cost per life-year saved
Base case	US$3,825
NMR Reduction	
10%	US$11,731
35%	US$3,352
High Coverage Comparison: 46% Intervention: 72%	US$3,221
Reduction in Costs (25%)	US$3,825
Functionality of SHGs (at 90%)	US$4,250

## Discussion and conclusion

Health interventions through Parivartan program at US$ 3,825 per life-year saved is within three times India’s per capita GDP of US$ 1,581. There are many social and health benefits of the intervention. A major limitation of this analysis and model is that it does not take into account all the health benefits of this intervention to both the mother and the newborn. We believe this intervention merits attention. The findings need to be contextualized keeping in view that this program was implemented as an innovation in the basket of high-intensity, high-input demonstration pilots, and was restricted to working with only about 224,287 women.

Self-help groups are primarily established with the goal of addressing financial needs, livelihood and productivity of women in Bihar. Initial results from the Parivartan program in Bihar suggest that SHGs are a useful platform to integrate maternal and child health interventions and also to broaden their utility beyond microfinance, particularly when they operate at a larger scale [19]. Evidence from this innovation suggests that this initiative has generated increased awareness about the importance of antenatal care, institutional deliveries, and childcare practices. This has important implications for not only scaling up maternal and child health interventions but also for addressing other priority health issues.

SHGs can be an effective platform to increase uptake of women’s health interventions and follow-up care. Early signs of better uptake of maternal and child health services (MCH) are observed where accredited social health activists (ASHA) are part of SHGs. These findings need to be further explored for improving efficiency of our front-line health workers. The SHG platform can be expanded beyond the safe motherhood program. With national scaling up of the proposed National Health Protection scheme, SHGs can play an important role in community monitoring, providing information about entitlements, and thereby improving utilization of health care services and reduction in the burden of catastrophic health expenditure.

The Government of India, under the National Urban Health Mission, has already adapted Mahila Arogya Samiti (Women’s health groups) to include community awareness, interpersonal communication, community-based monitoring and linkages with the services and referral. Similarly, the SHG initiative needs to be subsumed under the National Rural Livelihood Mission and the National Health Mission to leverage the existing platform for better health and productivity of the population.

This study contributes to closing an important evidence gap in cost-effectiveness of programs mobilizing women into self-help groups as a mechanism for layering health behavior change communication. Following the lessons from the Parivartan program, the Government of Bihar decided to scale up the health layering intervention across many districts of the state. The pathways to change and the hypothesis around mechanisms that make this intervention successful are ripe for further investigation. There is a need for stronger methodological solutions to capture the range of societal benefits arising out of the program.

## References

[1] Lorenzetti L. M., Leatherman S., & Flax V. L. (2017). Evaluating the effect of integrated microfinance and health interventions: an updated review of the evidence. Health policy and planning, 32(5), 732–756. 10.1093/heapol/czw170 28453714

[2] Gakidou E., Cowling K., Lozano R., & Murray C.J. (2010). Increased educational attainment and its effect on child mortality in 175 countries between 1970 and 2009: a systematic analysis. The Lancet, 376(9745), 959–974. 10.1016/S0140-6736(10)61257-3 20851260

[3] Murray C.J. & Lopez A.D. (1997). Alternative projections of mortality and disability by cause 1990–2020: Global Burden of Disease Study. The Lancet, 349(9064), 1498–1504. 10.1016/S0140-6736(96)07492-2 9167458

[4] Prost A, Colbourn T, Seward N, Azad K, Coomarasamy A, Copas A, et al (2013) Women's groups practising participatory learning and action to improve maternal and newborn health in low-resource settings: a systematic review and meta-analysis. The Lancet 381: 1736–1746. 10.1016/S0140-6736(13)60685-6 PMC3797417 23683640

[5] Rai RK (2014) Tracking women and children in a Continuum of Reproductive, Maternal, Newborn, and Child Healthcare (RMNCH) in India. Journal of epidemiology and global health 4: 239–243. 10.1016/j.jegh.2013.12.006 25107660 PMC7333814

[6] Devika J, Thampi BV (2007) Between ‘Empowerment’ and ‘Liberation’: The Kudumbashree Initiative in Kerala. Indian Journal of Gender Studies 14: 33–60.

[7] Saha S, Annear P, Pathak S (2013) The effect of Self-Help Groups on access to maternal health services: evidence from rural India. International journal for equity in health 12: 36 10.1186/1475-9276-12-36 23714337 PMC3673812

[8] Harper M. (2002). Promotion of self help groups under the SHG bank linkage program in India; In Seminar on SHG-bank linkage programme at New Delhi (pp. 25–26). Accessible from https://pdfs.semanticscholar.org/37e9/60be6d6bea3fe4b 5e718cbf0f3aa80efeb5b.pdf.

[9] Tankha A (2002) Self-help groups as financial intermediaries in India: Cost of promotion, sustainability and impact. New Delhi, available at www apmas org SME Technical Working Papers Series.

[10] Isern, J. (2007). Sustainability of self-help groups in India: Two analyses. Consultative group to assist the poor (CGAP). No. 12, August 2007. Available from: https://www.cgap.org/sites/default/files/researches/documents/CGAP-Occasional-Paper-Sustainability-of-Self-Help-Groups-in-India-Two-Analyses-Aug-2007.pdf.

[11] AccessAfrica (2011): Microfinance in Africa: State of the Sector Report 2011. Closing the Gap. Available from: https://www.care.org/sites/default/files/documents/MF-2011-CARE-Access-Africa-Closing-the-Gap.pdf.

[12] Darmstadt G. L., Bhutta Z. A., Cousens S., Adam T., Walker N., De Bernis L., & Lancet Neonatal Survival Steering Team. (2005). Evidence-based, cost-effective interventions: how many newborn babies can we save? The Lancet, 365(9463), 977–988. 10.1016/S0140-6736(05)71088-6 15767001

[13] Bhutta ZA, Das JK, Bahl R, Lawn JE, Salam RA, Paul VK, et al (2014) Can available interventions end preventable deaths in mothers, newborn babies, and stillbirths, and at what cost? The Lancet 384: 347–370. 10.1016/S0140-6736(14)60792-3 24853604

[14] Drummond M. F., Sculpher M. J., Claxton K., Stoddart G. L., & Torrance G. W. (2015). Methods for the economic evaluation of health care programmes Oxford: Oxford University Press.

[15] Haddix AC, Teutsch SM, Corso PS (2003) Prevention effectiveness: a guide to decision analysis and economic evaluation. Oxford: Oxford University Press.

[16] Varghese B, Peterman TA, Holtgrave DR (1999) Cost-effectiveness of counseling and testing and partner notification: a decision analysis. AIDS 13: 1745–1751. 10509577 10.1097/00002030-199909100-00019

[17] General R. (2013). Annual Health Survey 2012–13 Fact Sheet, Bihar. Office of Registrar General & Census Commissioner, India, Ministry of Home Affairs, Government of India Available from: http://www.censusindia.gov.in/vital_statistics/AHSBulletins/AHS_ Factsheets_2012-13/FACTSHEET-Bihar.pdf.

[18] Saggurti N., Irani L., (2018). Evaluating the impact of the Parivartan project in improving maternal and child health behaviors among women in self-help groups in Bihar, Accessible from: 10.7910/DVN/WPHXJK, Harvard Dataverse, V1.

[19] Saggurti N., Atmavilas Y., Porwal A., Schooley J., Das R., Kande N., et al (2018). Effect of health intervention integration within women's self-help groups on collectivization and healthy practices around reproductive, maternal, neonatal and child health in rural India. PLoS One, 13(8). 10.1371/journal.pone.0202562 PMC6107172 30138397

